# Clustering of extreme values: estimation and application

**DOI:** 10.1007/s10182-023-00474-y

**Published:** 2023-03-31

**Authors:** Marta Ferreira

**Affiliations:** Centro de Matemática, Universidade do Minho, Braga, Portugal

## Abstract

The extreme value theory (EVT) encompasses a set of methods that allow inferring about the risk inherent to various phenomena in the scope of economic, financial, actuarial, environmental, hydrological, climatic sciences, as well as various areas of engineering. In many situations the clustering effect of high values may have an impact on the risk of occurrence of extreme phenomena. For example, extreme temperatures that last over time and result in drought situations, the permanence of intense rains leading to floods, stock markets in successive falls and consequent catastrophic losses. The extremal index is a measure of EVT associated with the degree of clustering of extreme values. In many situations, and under certain conditions, it corresponds to the arithmetic inverse of the average size of high-value clusters. The estimation of the extremal index generally entails two sources of uncertainty: the level at which high observations are considered and the identification of clusters. There are several contributions in the literature on the estimation of the extremal index, including methodologies to overcome the aforementioned sources of uncertainty. In this work we will revisit several existing estimators, apply automatic choice methods, both for the threshold and for the clustering parameter, and compare the performance of the methods. We will end with an application to meteorological data.

## Introduction

**Fig. 1:**
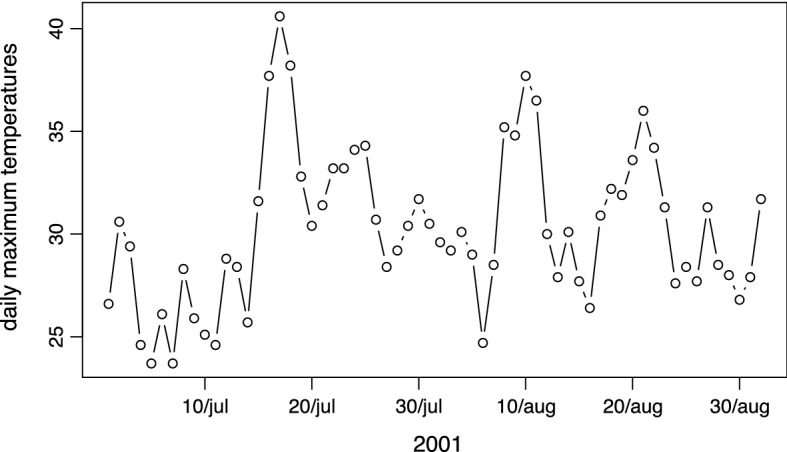
Daily maximum temperature in the months of July and August 2001 at the station Abrantes in the center of Portugal

Climate change is at the order of the day, leading to a growing concern about the occurrence of phenomena such as extreme drought, floods, and large-scale forest fires. The pandemic situation caused by COVID-19 and the outbreak of armed conflicts as happened recently also have a strong impact on the global society and economy in which we live. Perhaps we have never seen an occurrence of extreme phenomena like today and a consequent demand for the use of appropriate tools to assess their impact, such as those provided by the extreme values theory (EVT). The clustering effect of high values has a strong impact on the assessment of the risk associated with extreme phenomena and its main actor is the extremal index, denoted by $\theta$. Indeed, $\theta$ describes and quantifies the clustering amount of the extreme values in many stationary time series. In Fig. 1 we can see the daily maximum temperatures collected at the climatologic station Abrantes in center of Portugal (in Celsius degrees). Clustering of extreme values is visible and thus the presence of extremal local dependence. Recently, other areas such as Dynamical Systems have also been applying the concept of extremal index (Moloney et al. 2019; Freitas et al. 2021, among others).

Let $\pmb {X}=\{X_n\}_{n\ge 1}$ be a stationary sequence of random variables (r.v.) with common marginal distribution function (d.f.) *F*. We say that $\pmb {X}$ has extremal index $\theta \in [0,1]$ if for each $\tau >0$ there exists a sequence of normalized levels $u_n$, i.e., $n(1-F(u_n))\rightarrow \tau$, as $n\rightarrow \infty$, such that $P(M_{n}\le u_n)\rightarrow \exp (-\theta \tau )$, where $M_{n}=\max (X_1,...,X_n)$. If $\theta =1$ then the tail behavior of $\pmb {X}$ resembles an iid sequence, whenever $\theta <1$ leads to the occurrence of clusters of extreme values.

One extremal local dependence condition that is usually considered is the $D(u_n)$, which is basically a standard mixing condition that limits the long-range dependence at large values. It implies that any two exceedances of large $u_n$, $X_i>u_n$ and $X_j>u_n$, for sufficiently separated time points *i* and *j* are asymptotically independent (see, Leadbetter 1974).

If $\pmb {X}$ satisfies $D(u_n)$, we have $P(M_{n}\le u_n)\approx F^{n\theta }(u_n)$, for large *n* and $u_n$. Moreover, if there exist normalizing real constants $a_n>0$ and $b_n$ such that $F^n(a_nx+b_n)\rightarrow G(x)$, then *G* is the d.f. of a generalized extreme value distribution (GEV) and $P(M_{n}\le a_nx+b_n)\rightarrow H(x)\equiv G^\theta (x)$. If we consider $\{X_n^*\}_{n\ge 1}$ an iid sequence with the same marginal d.f. *F* of $\pmb {X}$, the limiting GEV distribution of the corresponding result for $M_{n}^*=\max (X_1^*,...,X_n^*)$ is $G(x)=H^{1/\theta }(x)$. In this context, $\theta$ plays a key role on the sample maxima distribution. The location and scale parameters of the GEV d.f. *H* and *G* are respectively related as follows: $\mu _H=\mu _G+\sigma _G(\theta ^\xi -1)/\xi$ and $\sigma _H=\sigma _G\theta ^\xi$, where the shape parameter $\xi$ is the same in both *G* and *H*. Thus ignoring $\theta$ may lead to misspecified tail inferences: underestimation of quantiles of *F* if inference is based on *H* from sample block maxima or overestimation of *H* quantiles if inferences are based on marginal *F* from sample observations (see Beirlant et al. 2004). The extremal index also corresponds to the reciprocal of the mean cluster size in the point process of exceedance times of a large threshold $u_n$, under a suitable mixing condition slightly stronger than $D(u_n)$ (Hsing et al. 1988). Another interpretation of $\theta$ due to O’Brien (1987) is based on a conditional probability that quantifies to what extent extremes cluster together.

Many contributions on the extremal index estimation are addressed in the literature, based on different interpretations of $\theta$. The major discussion focuses on strategies for the best choice of one or more auxiliary parameters involved in each method and respective stability of the estimates. Typically, the estimation of $\theta$ involves two sources of uncertainty: a threshold and some clustering parameter. Classical methods proposed in, e.g., Hsing (1991), Hsing (1993), Smith and Weissman (1994) and Weissman and Novak (1998) require both the choice of a threshold and a parameter associated with the clusters identification (runs parameter). More recently, threshold-dependent estimators have been proposed which are defined from the interexceedance times of a high threshold. This approach is based on the compound Poisson character of the point process of exceedances, namely, with an appropriate normalization and under a suitable and not restrictive mixing condition, the interexceedance times follow approximately an exponential mixture distribution with a point mass at zero and involving a parameter corresponding to $\theta$. These include the intervals estimator of Ferro and Segers (2003), the *K*-gaps estimator of Süveges (2010), the censored and the truncated estimators of (respectively, Holěsovský and Fusek 2020; Holesovsky and Fusek 2022). The cycles estimator of Ferreira and Ferreira (2018) also requires the choice of a threshold and the validity of a local dependence condition describing the cluster behavior. The maxima estimators of Gomes (Gomes 1993; Ancona-Navarrete and Tawn 2000; Northrop 2015) are based on the comparison between the block maxima distribution obtained from the stationary sequence and the corresponding sequence of independent variables. We also include in this group the more recent estimator of Ferreira and Ferreira (2022) which is obtained from the bivariate block maxima distribution generated from the stationary sequence and an iid sequence of independent standard Fréchet variables. These estimators require the choice of a block size. In this paper we introduce a new version of the block maxima estimator in Ferreira and Ferreira (2022) and propose a bootstrap method to compute confidence intervals. In Sect. 2 we present a survey on the inferential methods that will be used. We are going to analyze their performance trough simulation in Sect. 3. Besides the pointwise estimation we also address interval estimation mainly based on bootstrap. In Sect. 4 we will illustrate the methods on a climatological dataset. We end with a discussion in Sect. 5.

## Some characterization and estimation of $\theta$

In this section we describe some probabilistic characteristics of the extremal index that inspired the methodology and the mathematical expression of the estimators to be presented. These will be analyzed on the next section through simulation and their performances will also be compared.

We start with classical runs estimator that is related to the O’Brien (1987) characterization,

1 $$\begin{aligned} \theta =\lim _{n\rightarrow \infty }P(M_{1,r_n}\le u_n|X_1>u_n), \end{aligned}$$

where $r_n=o(n)$ and $M_{i,j}=\max (X_{i+1},..,X_{j})$, $i\le j-1$, with $M_{i,j}=-\infty$ if $i>j-1$ and $M_{0,j}\equiv M_{j}$. The empirical counterpart of (1) leads to the runs estimator

2 $$\begin{aligned} {\tilde{\theta }}^{(R)}=\frac{\sum _{i=1}^{n-r+1}\mathbbm {1}_{\{X_i>u,X_{i+1}\le u,...,X_{i+r-1}\le u\}}}{N_u} \end{aligned}$$

where $N_u$ denotes the number of exceedances of threshold *u* in stationary sequence $\pmb {X}$ and $\mathbbm {1}_{\{\cdot \}}$ denotes an indicator function (Hsing 1993). In practice, clusters are identified by considering two different groups of exceedances of a threshold *u* as independent clusters if there are at least $r-1$ consecutive observations below the threshold between them. We shall denote *r* the runs parameter.

Hsing and McCormick (1991) establishes a similar result to that of O’Brien, under a local mixing condition denoted $D^{(s)}(u_n)$ which states that within a cluster, an exceedance of a high threshold $u_n$ is most likely to be followed by another exceedance within $s-1$ consecutive observations. Condition $D^{(s)}(u_n)$ requires the validity of mixing condition $D(u_n)$ that limits the long-range dependence at extreme levels by implying that any two exceedances of $u_n$ that are sufficiently separated in time are asymptotically independent. More precisely, $\pmb {X}$ satisfies condition $D(u_n)$ if for any integers $1\le i_1<...< i_q< j_1<... <j_{q'}\le n$ for which $j_1-i_q\ge l$, we have

$$\begin{aligned} \left| P\left( M_{i_1,i_q}\le u_n,M_{j_1,j_{q'}}\le u_n\right) -P\left( M_{i_1,i_q}\le u_n\right) P\left( M_{j_1,j_{q'}}\le u_n\right) \right| \le \alpha _{n,l}, \end{aligned}$$

with $\alpha _{n,l_n}\rightarrow 0$, as $n\rightarrow \infty$, for some sequence $l_n=o(n)$ and $l_n\rightarrow \infty$.

Condition $D^{(s)}(u_n)$ will hold for $\pmb {X}$ if $D(u_n)$ also holds and there exists $s>0$ integer, sequences $r_n$ and $l_n$ of integers such that $r_n\rightarrow \infty$, $n\alpha _{n,l_n}/r_n\rightarrow 0$, $l_n/r_n\rightarrow 0$ and

$$\begin{aligned} \lim _{n\rightarrow \infty }nP\left( X_1>u_n\ge M_{1,s},M_{s,r_n}> u_n\right) =0. \end{aligned}$$

It is easily seen that once condition $D^{(s)}(u_n)$ holds, then $D^{(s^*)}(u_n)$ also holds for all $s^* \ge$s.

Consider $u_n(\tau )$ such that, for $\tau >0$,

$$\begin{aligned} \lim _{n\rightarrow \infty }nP\left( X_1>u_n(\tau )\right) =\tau . \end{aligned}$$

If $D^{(s)}(u_n)$ holds for the stationary sequence $\pmb {X}$, for some $s>0$ and $u_n=u_n(\tau )$ for all $\tau >0$, the extremal index $\theta$ of $\pmb {X}$ exists if and only if

3 $$\begin{aligned} \lim _{n\rightarrow \infty } P\left( M_{1,s}\le u_n|X_1>u_n\right) =\theta , \end{aligned}$$

for all $\tau >0$ (Hsing and T., McCormick, W.P. 1991, Corollary 1.3).

Observe that the runs estimator (2) also corresponds to the empirical counterpart of (3) by considering the runs parameter $r=s$. When $r=2$ we derive the Nandagopalan (1990) estimator which requires condition $D^{(2)}(u_n)$ to hold. Its formula is very easy to compute since it is simply the ratio between the number of upcrossings (or downcrossings) and the number of exceedances of $u_n$. The approach in Ferreira and Ferreira (2018) is based on the Nadagopalan’s estimator through an estimation procedure of the extremal index of an auxiliary stationary sequence satisfying $D^{(2)}(u_n)$. More precisely, if $\pmb {X}$ satisfies $D^{(s)}(u_n)$, we take the so called cycles process $\{Z_n=M_{(n-1)(s-1),n(s-1)}\}_{n\ge 1}$ for which $D^{(2)}(u_n)$ holds and estimate $\theta$ as the ratio between the number of upcrossings of threshold *u* within $\{Z_1,...,Z_{[n/(s-1)]}\}$, denoted $U_u^{Z}$ and the number of exceedances $N_u$ of $\pmb {X}$ (Ferreira and Ferreira 2018, Proposition 2.3), i.e.,

4 $$\begin{aligned} {\tilde{\theta }}^{(C)}=\frac{U_u^{Z}}{N_u}, \end{aligned}$$

here denoted cycles estimator.

Consider the interexceedance times r.v. $T(u_n)=\min \{j\ge 1:\, X_{j+1}>u_n|X_1>u_n\}$. Under a suitable mixing condition, we have that $P(X_1>u_n)T(u_n)$ converges in distribution to a mixture distribution which is degenerated at zero with weight $1-\theta$ and has exponential law with mean value $1/\theta$ with weight $\theta$. Therefore, the extremal index $\theta$ expresses both the proportion of intra-cluster (within a cluster) times and inter-cluster (between clusters) times, and the expected value of the inter-cluster times under a convenient normalization. The intervals estimator of Ferro and Segers (2003) corresponds to a moment-based estimator derived from the limiting mixture distribution. It only requires the choice of a high threshold exempting the choice of a runs parameter. It will be denoted ${\tilde{\theta }}^{(I)}$.

Although interexceedance times, $T_i=j_{i+1}-j_i$, $i=1,...,N_u$, are not independent and a likelihood procedure assumes independence, this assumption may be disregarded under the validity of condition $D^{(s)}(u_n)$ for some *s* (Süveges 2007; Süveges 2010). On the other hand, the normalized intra-cluster times are theoretically zero in the limit but they are observed as positive values and thus will be assigned to the exponential part of the limiting mixture law.

By considering the new r.v. *K*-gap $S^{(K)}(u_n) = \max (T(u_n)-K, 0)$, the smallest times $T_i$ are set to zero, which improves the identification of intra-cluster times. Süveges (2010) shows that the maximum likelihood (ML) method can be applied to the limit mixture model in order to estimate $\theta$, replacing interexceedance times by *K*-gaps $S^{(K)}$ and under condition $D^{(s)}(u_n)$ for $s=K+1$. In Süveges (2007), it was only addressed the case $K = 1$. This method corresponds to the so called *K*-gaps estimator, here denoted ${\tilde{\theta }}^{(K)}$. Choosing *K* involves some care, since if too large leads to more null times and consequent assignment to the degenerate part of the limiting mixture law, while too small *K* means fewer null times and hence an assignment tendency to the exponential component of the mixture law.

The censored estimator introduced in Holěsovský and Fusek (2020) is similar to the *K*-gaps estimator but the smallest interexceedance times (less than some integer *c*) are censored. The choice of *c* is not so sensitive as the choice of *K*, however, the likelihood expression underlying the censored estimator does not allow an explicit formula for it, which makes its analysis and improvements difficult. A new approach is considered in Holesovsky and Fusek (2022), where the extremal index estimation is based on truncation. The small interexceeding times are truncated by a given parameter *t*, corresponding to *K* in the *K*-gaps estimator or *c* in the censored estimator. If condition $D^{(s)}(u_n)$ holds for $\pmb {X}$ with $s=t+1$, under the same mixing condition considered to derive the limiting mixture law of $P(X_1>u_n)T(u_n)$, Holesovsky and Fusek (2022) prove that $P(X_1>u_n)(T(u_n)-t)|T(u_n)>t$ converges in distribution to an exponential law with expected value $1/\theta$. Let $T_{(1)}\le ...\le T_{(N_u-1)}$ be the order statistics of $T_1,...,T_{N_u-1}$, assume that $T_{(N-N_t-1)} \le t < T_{(N-N_t)}$, with $N_t$ the number of times that are greater than some fixed positive *t*, and $\{S_1,..., S_{N_t}\} = \{T_{(N-N_t)}-t,...,T_{(N-1)}-t\}$ the set of exceedance times above the truncation value *t*. The ML method can be applied since truncated times are not affected by the sequence dependence (inter-cluster times are asymptotically independent (Ferro and Segers 2003) and intra-cluster times have not propensity to exceed *t* under local dependence condition $D^{(t+1)}(u_n)$). A simple ML estimator for $\theta$ corresponds to the arithmetic inverse of sample mean

5 $$\begin{aligned} {\tilde{\theta }}=\frac{nN_t}{N_u\sum _{i=1}^{N_t}S_i} \end{aligned}$$

The derivation of the bias of (5) and of a penultimate approximation of the limiting distribution leads to an improved and bias corrected estimator, yielding the so-called truncated estimator

6 $$\begin{aligned} {\tilde{\theta }}^{(T)}={\tilde{\theta }}^{BC}-\frac{N_u}{2n(N_u-1)}\left[ 1+ {\tilde{\theta }}^{BC}(N_u-4)-\left( {\tilde{\theta }}^{BC}\right) ^2(N_u-1)\right] , \end{aligned}$$

with

$$\begin{aligned} {\tilde{\theta }}^{BC}= \frac{(N_u-1){\tilde{\theta }}-1}{N_u-1+(N_u/n)t}, \end{aligned}$$

where ${\tilde{\theta }}$ is given in (5).

In applications, it can be difficult to check the validity of $D^{(s)}(u_n)$ condition. Various proposals have been presented, such as diagnostic plots of anti-$D^{(s)}(u_n)$ (Süveges 2007; Ferreira and Ferreira 2018), the information matrix test (Süveges 2010; Fukutome and Süveges 2014; Fukutome et al. 2019), or based on a stability check of the runs estimator (Cai 2019). However, the study of this issue is not closed and still awaits developments. The automation procedure in Fukutome and Süveges (2014); Fukutome et al. (2019) allows to select both *s* and $u_n$. More precisely, considering the *K*-gaps estimator, it is based on misspecification tests through the information matrix test (IMT) presented in ( Süveges 2010). All combinations of pairs of thresholds and run parameters in plausible ranges are tested for misspecification of the model, and the pair (*u*, *K*) that generates the largest number of observations after declustering, within a list of pairs of small misspecification (IMT < 0.05) is selected, provided the number of exceedances is larger than 80. We will apply this automation procedure to select both threshold and runs parameter involved in each of the estimators: runs ${\tilde{\theta }}^{(R)}$, cycles ${\tilde{\theta }}^{(C)}$, intervals ${\tilde{\theta }}^{(I)}$, *K*-gaps ${\tilde{\theta }}^{(K)}$ and truncated ${\tilde{\theta }}^{(T)}$, in order to compare their performances through simulation and further on the analysis of real data. For a given selected pair (*u*, *K*), we will assume the validity of condition $D^{(K+1)}(u)$. In the case of the intervals estimator, only the IMT threshold selection will be used since it solely depends on the threshold choice.

There are other estimation methods of $\theta$ entailing the choice of a single tuning parameter. Maxima methods as described in Gomes (Gomes 1993; Ancona-Navarrete and Tawn 2000; Northrop 2015) and more recently in Ferreira and Ferreira (2022) require the choice of a block length in order to generate a block maxima sequence from the original one. The two first references present methods that need to resample the original data to produce a sample of block maxima with approximate d.f. *G*. Northrop proposal in Northrop (2015) avoids this drawback by comparing the limiting GEV *H* of the maxima $M_n$ of $\pmb {X}$ directly to the marginal d.f. *F*. More precisely, for large enough *n*, we have $H\approx F^{n\theta }$, thus $Y=-n\log F(M_n)$ has exponential law with mean value $1/\theta$. The Northrop estimator is based on the ML approach, considering the sample of block maxima $\{M_{(i-1)b,ib},\,i=1,...,[n/b]\}$ of *b* consecutive values of $\{X_1,...,X_n\}$ and estimating the unknown d.f. *F* by the respective empirical d.f.. This corresponds to the disjoint blocks estimator. The sliding blocks version of Northrop estimator is based on a sample of overlapping block maxima $\{M_{i-1,i+b-1},\,i=1,...,n-b+1\}$, leading to

7 $$\begin{aligned} {\tilde{\theta }}^{(N)}=\left( \frac{1}{n-b+1}\sum _{i=1}^{n-b+1}Y_i\right) ^{-1}, \end{aligned}$$

with $Y_i=-b\log {\hat{F}}(M_{i-1,i+b-1})$, $i=1,...,n-b+1$. We will use the sliding blocks ${\tilde{\theta }}^{(N)}$ which compares favorably with the disjoint blocks (Northrop 2015). This will be denoted the Northrop estimator.

In Ferreira and Ferreira (2022) a new block maxima method was introduced to estimate the extremal index. It is based on a bivariate sequence $\{(Y_{n,1}={X}^{*}_n,Y_{n,2}=(1/2){X}^{*}_n\vee (1/2)X_n)\}_{n}\,$ generated from the original $\pmb {X}$ which is assumed to have standard Fréchet marginals and by an i.i.d. sequence $\{{X}^{*}_n\}_{n}$ also having standard Fréchet marginals. Operator $\vee$ stands for “maximum". It is proved that the component-wise maxima of sequence $\{(Y_{n,1},Y_{n,2})\}_{n}.$ has a limiting bivariate extreme value copula $C(u,v)=\min (uv^{\frac{\theta }{1+\theta }},v)$ with tail dependence coefficient

8 $$\begin{aligned} \lambda =\displaystyle \lim _{v\rightarrow 1^{-}}2-\frac{1-C(v,v)}{1-v} =\frac{1}{1+\theta }. \end{aligned}$$

leading to estimator

9 $$\begin{aligned} {\widetilde{\theta }}=\frac{1}{{\widetilde{\lambda }}\vee 1/2}-1. \end{aligned}$$

**Algorithm**: The estimation procedure follows the steps below:

Step 1. Take the marginal transformation $-\frac{1}{\log {\widetilde{F}}_{X}(X_i)}$, where ${\widetilde{F}}_{X}$ is an empirical d.f. of sample $X_1,...,X_n$ with dimension *n*, in order to have approximately standard Fréchet marginals.
Step 2. Generate an i.i.d. sequence with standard Fréchet d.f., ${X}^*_1,...,{X}^*_n$, and take $({X}^*_i,(1/2){X}^*_i\vee (1/2)X_i)$, $i=1,...,n$.
Step 3. Choose the blocks length *b* to generate a bivariate sample of component-wise maxima, and estimate $\lambda$ (see Ferreira and Ferreira 2022 and references therein for details). Here we consider sliding blocks ${\left( Z_{j,1},Z_{j,2}\right) =}\left( \bigvee _{i=j}^{j+b-1}{X}^*_i,\bigvee _{i=j}^{j+b-1}(1/2){X}^*_i\vee (1/2)X_i\right) ,\,1\le j\le n-b+1$.
Step 4. Calculate ${\widetilde{\theta }}$ in (9).
Step 5. Repeat steps 2–4 a large number *R* of times, obtain estimates ${\widetilde{\theta }}_{1},...,{\widetilde{\theta }}_{R}$ and estimate
  10 $$\begin{aligned} {\tilde{\theta }}^{(F)}=\frac{1}{R}\sum _{j=1}^{R}{\widetilde{\theta }}_{j} \end{aligned}$$
  in order to achieve robustness given the existence of arbitrariness in the generation of a random sample (Step 2) in each estimate.

Step 4 is based on estimator (9 which requires an estimate of the tail dependence coefficient $\lambda$. We follow the proposal of Ferreira and Ferreira (2022) with

$$\begin{aligned} {\widetilde{\lambda }}=3-\frac{1}{1-\frac{1}{n}\sum _{i=1}^{n}\left( {\widetilde{G}}_1(Z_{i,1})\vee {\widetilde{G}}_2(Z_{i,2})\right) }, \end{aligned}$$

where ${\widetilde{G}}_j$, $j=1,2$, is an empirical distribution function of the GEV marginal $G_j$ of $Z_{1,j}$. For more details, see Ferreira and Ferreira 2022. In Ferreira and Ferreira (2022) it was considered $R=10000$ and block maxima taken on disjoint blocks. Here we consider the sliding blocks approach as in the Northrop estimator. Some prior simulations lead us to the proposal that $R=100$ is reasonable for a robustness of the method. This approach will be denoted Ferreira estimator.

In the following we provide a catalog of some distributions and their associated extremal indices. Additional lists of models and respective extremal indices are also exposed in Gomes and Neves (2015, 2020).

- A first order auto-regressive with maximum operator (MAR), $X_i=\max (\phi X_{i-1},\epsilon _i)$, $i\ge 1$, $X_0=\epsilon _1/(1-\phi )$, $\{\epsilon _i\}$ i.i.d. with standard Fréchet marginals (Davis and Resnick 1989), for which $\theta =1-\phi$. See, e.g., ( Leadbetter (1983)) and references therein;
- A moving maxima $X_i=\max _{j=0,...,d} a_jZ_{d-j}$ with $\{Z_i\}$ i.i.d. standard Fréchet (MMFrec), where parameters $a_j\ge 0$ and $\sum _{j=0}^{d} a_j=1$ (Deheuvels 1983), for which $\theta =\max _{j=0,...,d} a_j$ (see, e.g., Beirlant et al. (2004));
- A Markov chain with standard Gumbel marginals and 1-lag bivariate logistic dependence (MCBEV), $P(X_i\le x,X_{i+1}\le y)=\exp (-(x^{-1/\alpha }+y^{-1/\alpha })^{\alpha })$. Calculations of $\theta$ for particular cases are found in Smith (1992);
- An ARCH(1) process, $X_i=(\beta + \lambda X_{i-1}^2)^{1/2}\epsilon _i$, with i.i.d. standard Gaussian innovations $\{\epsilon _i\}$. (Embrechts et al. 1997) addresses the (not straightforward) extremal index computation of ARCH models;
- A first order auto-regressive with Cauchy standard marginals (ARCau), $X_i=\rho X_{i-1}+\epsilon _i$, $\{\epsilon _i\}$ i.i.d. having Cauchy d.f. with mean 0 and scale $1-|\rho |$. According to (Hsing and McCormick 1991), $\theta =1-\rho$ if $\rho \ge 0$ and $\theta =1-\rho ^2$ if $\rho <0$;
- A first order auto-regressive negatively correlated uniform (ARUnif), $X_i=-(1/r) X_{i-1}+\epsilon _i$, $i\ge 1$, $\{\epsilon _i\}$ i.i.d. where $P(\epsilon _1=k/r)=1/r$ for $k=1,...,r$, with $X_0\sim U(0,1)$ independent of $\epsilon _i$, having $\theta =1-1/r^2$
- An AR(1) process, $X_i=\phi X_{i-1}+\epsilon _i$, $i\ge 1$, $\{\epsilon _i\}$ i.i.d. *N*(0, 1), $X_0\frown N(0,1/(1-\phi ^2))$, with $\theta =1$;

These models will be used in the simulation study, in the next section.

## Simulation study

In this section we analyze the estimation of the extremal index through simulation. We compare the performances of the runs (R) estimator in (2), the cycles (C) estimator in (4), the truncated (T) estimator in (6), the intervals (I) estimator of Ferro and Segers (2003), the *K*-gaps (K) estimator of Süveges (2010), along with the block maxima estimators of Northrop (N) in (7) and Ferreira (F) in (10). The first five estimators require the selection of a runs parameter and a threshold, except the intervals estimator only needing the choice of a threshold. To this end we apply the IMT method ( Süveges 2010; Fukutome and M.A. Süveges M. 2014; Fukutome et al. 2019) described in Sect. 2. The block maxima estimators require the settlement of a block length and no automation procedure is considered. In the study we take block lengths $b=10,20,...,70$. The software( R Core Team 2020) was used and the R codes of estimators can be seen in https://github.com/msferreirauminho/msrf.

The simulation study is based on the following models: a first order max auto-regressive (MAR) process with standard Fréchet marginals and autoregressive parameter $\phi =0.5$ ( Davis and Resnick 1989) satisfying condition $D^{(2)}(u_n)$ (see, e.g.,Cai (2019)); a moving maxima (MMFrec) process with coefficients $a_j=1/5$, $j\in \{1,2,3,7,8\}$ ( Deheuvels 1983) for which $D^{(5)}(u_n)$ holds (Ferreira and Ferreira 2018); a Markov chain (MCBEV) with standard Gumbel marginals and logistic joint distribution with dependence parameter $\alpha =0.5$ (Smith 1992); an ARCH(1) process with Gaussian innovations, autoregressive parameter $\lambda =0.5$ and variance parameter $\beta =1.9\cdot 10^{-5}$ (Embrechts et al. 1997); an AR(1) process with Cauchy marginals and auto-regressive parameter $\rho =-0.6$ ( Chernick 1978) and a negatively correlated uniform AR(1) process with $r=2$ ( Hsing and McCormick 1991), respectively denoted ARCau and ARUnif and both satisfying condition $D^{(3)}(u_n)$. The extremal index values of the processes MAR, MMFrec, MCBEV, ARCH, ARCau and ARUnif are 0.5, 0.2, 0.328, 0.835, 0.64 and 0.75, respectively. We also consider a classical first order AR(1) process with Gaussian marginals and auto-regressive parameter 0.5, here denoted AR, which is almost independent and satisfies condition $D^{(1)}(u_n)$ having $\theta =1$. As far as we know, there is no theoretical analysis on condition $D^{(s)}(u_n)$ for MCBEV model and we mention an empirical evaluation conducted in Ferreira and Ferreira (2018). Cai (2019) proved that $D^{(s)}(u_n)$ doesn’t hold for a particular ARCH model.

**Fig. 2:**
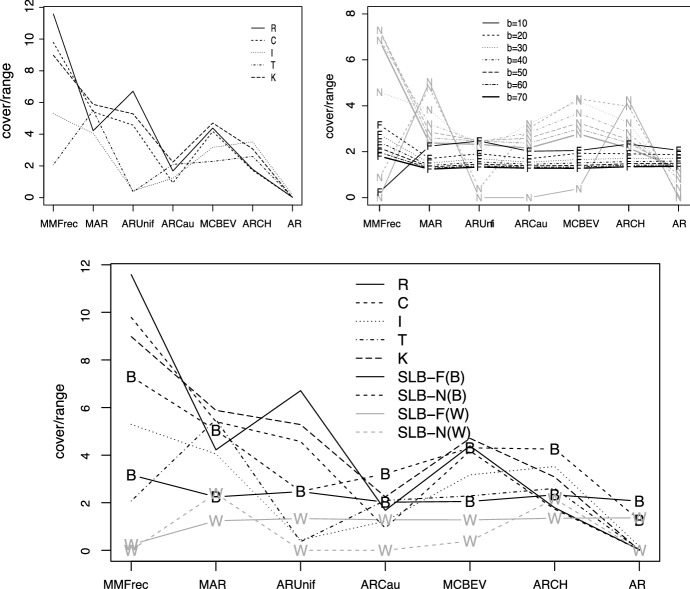
The ratio between the coverage proportion of the true extremal index value within the estimated intervals and the respective range width (“cover/range") obtained for: (left-top) the runs (R), the cycles (C), the intervals (I), the truncated (T) and the *K*-gaps (K); (right-top) the sliding blocks of ( Ferreira and Ferreira (2022)) (black) and of ( Northrop (2015), ) (grey), for block lengths $b=10,20,...,70$; (bottom) the runs (R), the cycles (C), the intervals (I), the truncated (T) and the *K*-gaps (K), the sliding blocks of ( Ferreira and Ferreira (2022)) (black) and of ( Northrop (2015)) (grey), for block lengths corresponding to the largest “cover/range", denoted by B (from best) and with the smallest “cover/range", denoted by W (from worst)

**Fig. 3:**
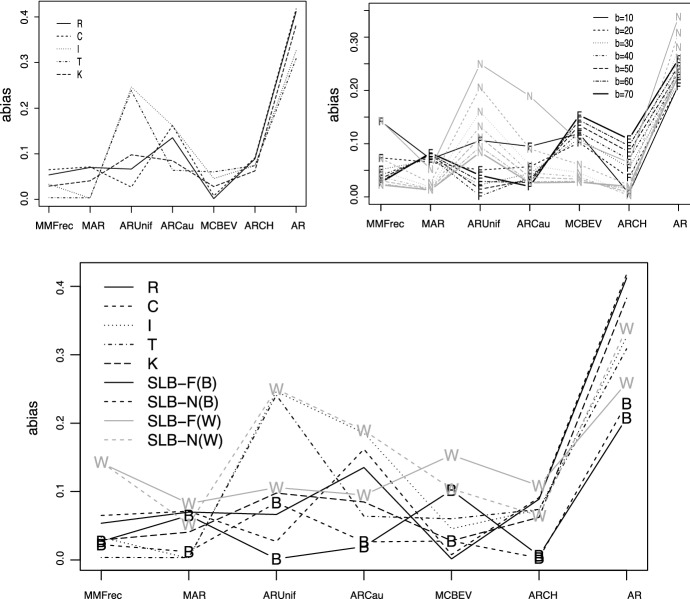
The absolute bias (abias) obtained for: (left-top) the runs (R), the cycles (C), the intervals (I), the truncated (T) and the *K*-gaps (K); (right-top) the sliding blocks of (Ferreira and Ferreira (2022)) (black) and of Northrop (2015) (grey), for block lengths $b=10,20,...,70$; (bottom) the runs (R), the cycles (C), the intervals (I), the truncated (T) and the *K*-gaps (K), the sliding blocks of Ferreira and Ferreira (2022) (black) and of Northrop (2015) (grey), for block lengths corresponding to the smallest “abias", denoted by B (from best) and with the largest “abias", denoted by W (from worst)

**Fig. 4:**
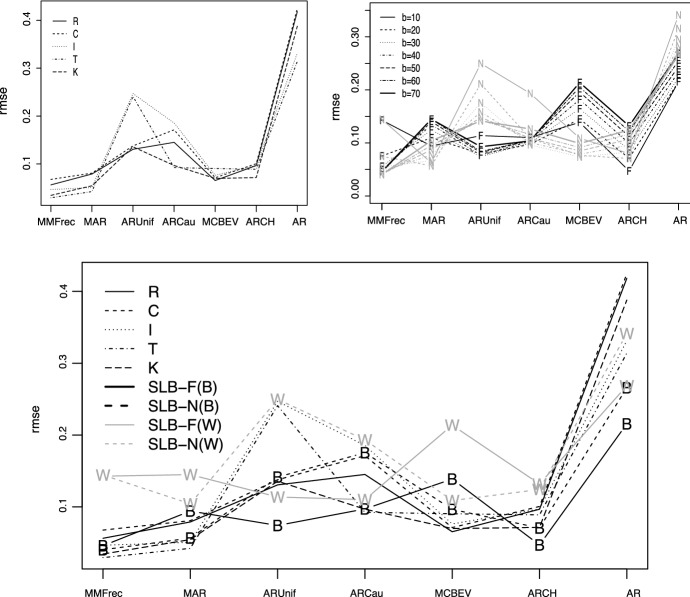
The root mean squared error (rmse) obtained for: (left-top) the runs (R), the cycles (C), the intervals (I), the truncated (T) and the *K*-gaps (K); (right-top) the sliding blocks of Ferreira and Ferreira (2022) (black) and of Northrop (2015) (grey), for block lengths $b=10,20,...,70$; (bottom) the runs (R), the cycles (C), the intervals (I), the truncated (T) and the *K*-gaps (K), the sliding blocks of Ferreira and Ferreira (2022) (black) and of Northrop (2015) (grey), for block lengths corresponding to the smallest “rmse", denoted by B (from best) and with the largest “rmse", denoted by W (from worst)

We consider 1000 replicas of each model and compute the mean, the absolute bias (abias), the root mean squared error (rmse) and the standard deviation (sd). Bootstrap confidence intervals were obtained through percentile method technique on time series. Confidence intervals were obtained through bootstrap percentile method technique on time series with fixed block length 20 ( Kunsch 1989; Politis and Romano 1994), except in the case of the intervals estimator where the bootstrap proposal in Ferro and Segers (2003) was used. In the case of the likelihood estimators (*K*-gaps and Northrop block maxima), the confidence intervals are based on the asymptotic Normality. In Ferreira sliding blocks estimator we considered percentiles 2.5 and 97.5 applied on the auxiliary estimates ${\widetilde{\theta }}_{j}$, $j=1,...,R$, produced by the method in Step 5 of the Algorithm in Sect. 2. We present the the proportion of intervals in the simulations that included the true value of $\theta$ (cov), the mean range width (rw) and the rate cov/rw corresponding to the proportion of coverage (cov) divided by the mean range width (rw). Some of the confidence intervals associated to Northrop sliding blocks failed to be computed because of the invalid standard error estimates which were accounted in column “NAs” of Table 2. The results for the runs, cycles, intervals, truncated and *K*-gaps estimators are presented in Table 1 and the estimates of Ferreira sliding blocks derived in (10) can be seen in Table 3. The rate cov/rw is plotted in Fig. 2 obtained for the runs, the cycles, the intervals, the truncated and the *K*-gaps estimators (left-top), for the Ferreira and Northrop sliding blocks estimators with block lengths $b=10,20,...,70$ (right-top) and the third bottom plot includes all estimators where for each of the sliding blocks estimators we address the best scenario (corresponding to the block length with the estimated largest rate, denoted by B) and the worst scenario (where the block length choice led to the smallest estimated rate, denoted by W). Analogous plots are represented in Figs. 3 and 4 relating to abias and rmse, respectively.

Cases closer to the border values of the extremal index domain were also considered, namely, MAR($\phi =0.9$) with $\theta =0.1$ and MAR($\phi =0.1$) with $\theta =0.9$. In the almost independence Gaussian AR model with $\theta =1$, besides parameter $\phi =0.5$ which induces strong dependence, we also analyze the AR weaker dependence models AR($\phi =0.1$) and AR($\phi =-0.1$) where $\theta$ is still unit but they are even more close of independence. See Tables 4, 5 and 6.

The cycles (C), the runs (R) and *K*-gaps (K) estimators present a somewhat homogeneous performance across the various models, with the *K*-gaps estimator slightly better behaved. We recall that we are applying the IMT method in all estimators, except in the sliding blocks, which was developed under the *K*-gaps estimator. On the other hand, the intervals (I) and the truncated (T) estimators show some sensitivity to the different models: they perform very well in the MMFrec, MAR, MCBEV and ARCH, but their biases and rmse are high in the ARUnif model.

We can also see that the choice of the block-maxima size to be used in sliding blocks estimators is important as their behavior improve in the best block choices. Indeed, both Ferreira and Northrop sliding blocks estimators present the best overall performances along the different models under the best block choices. Both Northrop and Ferreira sliding blocks have to deal with a trade-off between bias (larger for small block-maxima lengths) and variance (increases with the block-maxima length). This can be observed in Tables 2 and 3. In practice one can plot estimates for several block-maxima sizes *b* and choose the smallest *b* above which estimates are approximately constant (Northrop 2015).

In the coverage rate (Fig. 2), it is observed that the *K*-gaps estimator presents an overall better performance. Yet, the coverage percentages (“cov") in Table 1 are not as large as would be desirable. This is particularly notorious in cases where estimates present larger rmse like AR model. In order to analyze the effect of bootstrap block-size choice on CI coverage, we have also applied the automatic block-size choice method developed in Politis and White (2004) and (Patton et al. 2009), available in R package *blocklength* Stashevsky 2022. We recall that the bootstrap CI requiring block-size selection was considered for the runs, cycles and truncated estimators. The results of the automatic block-size choice method are in Table 7 where we observe improvements for the truncated estimator, except in the ARCH model. On the other hand, the ARCH model benefits from the automatic method within the runs and cycles estimators. The MAR model with $\theta =0.1$ also gains with the automated method. The Ferreira sliding blocks estimator presents both the highest coverage proportions (cov) and range widths (rw), leading to low coverage rates (“cov/rw"), as can be seen in Table 3 and Fig. 2. The compromise between both high coverage (“cov") and coverage rate (“cov/rw") points out the Northrop sliding blocks estimator as the best choice, particularly for larger block-maxima sizes.

The AR model with stronger dependence (i.e., dependence parameter $\phi =0.5$) presents the worst performance in all estimators (see, e.g., Figs. 2, 3 and 4). Both sliding blocks estimators seem to be the most promising in this model, which has an extremal index equal to one and, therefore, a boundary value in the support of $\theta$. However, the same is not true with weaker dependent AR models (i.e., with dependence parameter $\phi =\pm 0.1$), where the absolute bias and rmse are lower, particularly within intervals (I), truncated (T) and Northrop (N) estimators (Tables 4, 5 and 6). The bootstrap confidence intervals of runs (R) and cycles (C) exhibit very small coverage percentages as well as the ones of *K*-gaps estimator based on ML, for all considered AR models. In the challenging cases of $\theta$ with values close to the domain boundaries 0 and 1 through model MAR with autoregressive parameters $\phi =0.1$ and $\phi =0.9$, corresponding to $\theta =0.9$ and $\theta =0.1$, respectively, we observe that estimators of runs (R), cycles (C), *K*-gaps and Ferreira sliding blocks (F) tend to behave better for $\theta =0.1$ than $\theta =0.9$ while in the truncated (T) estimator the conclusion is opposite. The intervals (I) and Northrop sliding blocks (N) present an overall better performance in both situations.

**Table 1:** Simulation results obtained for the runs (R), cycles (C), intervals (I), truncated (T) and K-gaps (K): mean, absolute bias (abias), root mean squared error (rmse), standard deviation (sd), the coverage proportion of the true extremal index value within the estimated confidence intervals (cov), the intervals range width (rw) and the ratio between “cov" and “rw"

	Mean	Abias	Rmse	Sd	Cov	Rw	Cov/rw
*R*							
MMFrec	0.1463	0.0537	0.0564	0.0173	0.9630	0.0831	11.5854
MAR	0.4300	0.0700	0.0791	0.0369	0.6190	0.1466	4.2211
ARUnif	0.8165	0.0665	0.1307	0.1125	0.7070	0.1054	6.7102
ARCau	0.5048	0.1352	0.1452	0.0528	0.2750	0.1636	1.6811
MCBEV	0.3296	0.0016	0.0655	0.0655	0.8300	0.1891	4.3896
ARCH	0.7461	0.0889	0.0968	0.0383	0.2260	0.1264	1.7878
AR	0.5879	0.4121	0.4181	0.0704	0.0040	0.1576	0.0254
*C*							
MMFrec	0.1349	0.0651	0.0678	0.0188	0.8280	0.0846	9.7900
MAR	0.4287	0.0713	0.0809	0.0384	0.8000	0.1480	5.4070
ARUnif	0.7770	0.0270	0.1378	0.1352	0.5050	0.1104	4.5730
ARCau	0.4780	0.1620	0.1713	0.0558	0.1530	0.1638	0.9343
MCBEV	0.3202	0.0078	0.0713	0.0709	0.7590	0.1817	4.1780
ARCH	0.7438	0.0912	0.1007	0.0425	0.2200	0.1274	1.7263
AR	0.5822	0.4178	0.4245	0.0751	0.0040	0.1590	0.0252
*I*							
MMFrec	0.2332	0.0332	0.0467	0.0329	0.6500	0.1228	5.2913
MAR	0.4975	0.0025	0.0508	0.0508	0.9670	0.2388	4.0501
ARUnif	0.9963	0.2463	0.2470	0.0189	0.0130	0.0297	0.4373
ARCau	0.8012	0.1612	0.1856	0.0920	0.3190	0.2608	1.2234
MCBEV	0.3737	0.0457	0.0763	0.0611	0.8310	0.2622	3.1695
ARCH	0.9089	0.0739	0.0973	0.0633	0.7370	0.2093	3.5207
AR	0.6741	0.3259	0.3312	0.0588	0.0450	0.2745	0.1639
*T*							
MMFrec	0.2037	0.0037	0.0295	0.0293	0.2510	0.1211	2.0727
MAR	0.5035	0.0035	0.0424	0.0423	0.8910	0.1620	5.5000
ARUnif	0.9887	0.2387	0.2409	0.0319	0.0301	0.0833	0.3620
ARCau	0.7040	0.0640	0.0925	0.0669	0.4220	0.2012	2.0974
MCBEV	0.3883	0.0603	0.0905	0.0674	0.4670	0.2042	2.2870
ARCH	0.9090	0.0740	0.0890	0.0495	0.3980	0.1530	2.6013
AR	0.6914	0.3086	0.3140	0.0578	0.0050	0.1815	0.0275
*K-gaps*							
MMFrec	0.1708	0.0292	0.0345	0.0184	0.8340	0.0929	8.9819
MAR	0.4593	0.0407	0.0546	0.0364	0.7010	0.1192	5.8818
ARUnif	0.8478	0.0978	0.1358	0.0943	0.4410	0.0835	5.2834
ARCau	0.5552	0.0848	0.0969	0.0469	0.3020	0.1346	2.2444
MCBEV	0.3561	0.0281	0.0699	0.0640	0.5760	0.1224	4.7048
ARCH	0.7726	0.0624	0.0716	0.0351	0.3280	0.1065	3.0800
AR	0.6174	0.3826	0.3878	0.0636	0.0000	0.1300	0.0000

**Table 2:** Simulation results obtained for the Northrop (2015) sliding blocks estimator: mean, absolute bias (abias), root mean squared error (rmse), standard deviation (sd), the coverage proportion of the true extremal index value within the estimated confidence intervals (cov), the intervals range width (rw), the ratio between “cov" and “rw" and the number of replicates where the confidence intervals couldn’t be calculated (NAs)

	Mean	Abias	Rmse	Sd	Cov	Rw	Cov/rw	NAs
*N b=10*								
MMFrec	0.3431	0.1431	0.1444	0.0189	0.0000	0.0640	0.0000	0
MAR	0.5522	0.0522	0.0626	0.0345	0.6700	0.1329	5.0433	0
ARUnif	0.9998	0.2498	0.2498	0.0026	0.0000	0.0145	0.0000	356
ARCau	0.8294	0.1894	0.1933	0.0387	0.0000	0.1047	0.0000	15
MCBEV	0.4319	0.1039	0.1089	0.0326	0.0440	0.1134	0.3881	0
ARCH	0.8996	0.0646	0.0800	0.0471	0.6433	0.1512	4.2547	2
AR	0.6616	0.3384	0.3412	0.0432	0.0000	0.1748	0.0000	0
*N b=20*								
MMFrec	0.2721	0.0721	0.0750	0.0209	0.0655	0.0741	0.8836	7
MAR	0.5264	0.0264	0.0567	0.0502	0.9068	0.1896	4.7823	2
ARUnif	0.9557	0.2057	0.2113	0.0482	0.0482	0.1217	0.3958	66
ARCau	0.7303	0.0903	0.1084	0.0600	0.5859	0.1937	3.0249	5
MCBEV	0.3887	0.0607	0.0767	0.0468	0.7230	0.1679	4.3057	0
ARCH	0.8572	0.0222	0.0707	0.0671	0.8655	0.2181	3.9692	11
AR	0.6916	0.3084	0.3156	0.0672	0.0261	0.2677	0.0973	2
*N b=30*								
MMFrec	0.2489	0.0489	0.0545	0.0241	0.3777	0.0822	4.5925	15
MAR	0.5185	0.0185	0.0668	0.0642	0.9130	0.2389	3.8209	0
ARUnif	0.9101	0.1601	0.1755	0.0720	0.3922	0.1964	1.9966	31
ARCau	0.6991	0.0591	0.0966	0.0764	0.8248	0.2566	3.2147	1
MCBEV	0.3736	0.0456	0.0736	0.0579	0.8640	0.2052	4.2108	0
ARCH	0.8405	0.0055	0.0830	0.0829	0.8809	0.2707	3.2537	9
AR	0.7186	0.2814	0.2951	0.0888	0.1401	0.3265	0.4292	1
*N b=40*								
MMFrec	0.2374	0.0374	0.0462	0.0271	0.6214	0.0898	6.9219	86
MAR	0.5153	0.0153	0.0773	0.0758	0.8608	0.2689	3.2014	16
ARUnif	0.8827	0.1327	0.1582	0.0861	0.6002	0.2472	2.4276	117
ARCau	0.6852	0.0452	0.1008	0.0901	0.8301	0.2938	2.8257	35
MCBEV	0.3660	0.0380	0.0786	0.0689	0.8696	0.2362	3.6817	3
ARCH	0.8321	0.0029	0.0959	0.0959	0.8535	0.3018	2.8281	51
AR	0.7383	0.2617	0.2829	0.1075	0.2876	0.3559	0.8083	30
*N b=50*								
MMFrec	0.2302	0.0302	0.0419	0.0291	0.7029	0.0963	7.2989	145
MAR	0.5144	0.0144	0.0870	0.0859	0.8494	0.2971	2.8595	37
ARUnif	0.8618	0.1118	0.1482	0.0974	0.7037	0.2839	2.4785	119
ARCau	0.6769	0.0369	0.1085	0.1020	0.8348	0.3217	2.5945	62
MCBEV	0.3613	0.0333	0.0850	0.0783	0.8583	0.2638	3.2533	5
ARCH	0.8264	0.0086	0.1067	0.1064	0.8339	0.3296	2.5300	73
AR	0.7536	0.2464	0.2743	0.1206	0.3947	0.3838	1.0283	65
*N b=60*								
MMFrec	0.2250	0.0250	0.0399	0.0311	0.7695	0.1056	7.2873	37
MAR	0.5120	0.0120	0.0952	0.0945	0.8839	0.3398	2.6008	1
ARUnif	0.8443	0.0943	0.1424	0.1067	0.7638	0.3097	2.4662	39
ARCau	0.6685	0.0285	0.1163	0.1128	0.8688	0.3664	2.3715	9
MCBEV	0.3572	0.0292	0.0913	0.0866	0.8880	0.2964	2.9961	3
ARCH	0.8199	0.0151	0.1169	0.1160	0.8503	0.3567	2.3836	18
AR	0.7629	0.2371	0.2698	0.1288	0.5111	0.4172	1.2252	10
*N b=70*								
MMFrec	0.2225	0.0225	0.0404	0.0336	0.7870	0.1150	6.8446	136
MAR	0.5132	0.0132	0.1043	0.1035	0.8463	0.3550	2.3842	37
ARUnif	0.8338	0.0838	0.1415	0.1140	0.7731	0.3331	2.3209	114
ARCau	0.6665	0.0265	0.1263	0.1235	0.8318	0.3850	2.1607	49
MCBEV	0.3557	0.0277	0.0990	0.0951	0.8687	0.3135	2.7708	10
ARCH	0.8153	0.0197	0.1243	0.1227	0.8273	0.3828	2.1612	85
AR	0.7714	0.2286	0.2658	0.1357	0.5303	0.4194	1.2643	42

**Table 3:** Simulation results obtained for the ( Ferreira and Ferreira 2022) sliding blocks estimator: mean, absolute bias (abias), root mean squared error (rmse), standard deviation (sd), the coverage proportion of the true extremal index value within the estimated confidence intervals (cov), the intervals range width (rw), the ratio between “cov" and “rw" and the number of replicates where the confidence intervals couldn’t be calculated (NAs)

	Mean	Abias	Rmse	Sd	Cov	Rw	Cov/rw
*F b=10*							
MMFrec	0.3419	0.1419	0.1428	0.0155	0.0660	0.2725	0.2422
MAR	0.5711	0.0711	0.0943	0.0620	0.9930	0.4422	2.2454
ARUnif	0.8559	0.1059	0.1138	0.0416	1.0000	0.4055	2.4663
ARCau	0.7349	0.0949	0.1102	0.0561	0.9980	0.4937	2.0213
MCBEV	0.4467	0.1187	0.1388	0.0720	0.7090	0.3454	2.0528
ARCH	0.8287	0.0063	0.0471	0.0467	1.0000	0.4290	2.3312
AR	0.7899	0.2101	0.2156	0.0484	0.9470	0.4589	2.0637
*F b=20*							
MMFrec	0.2733	0.0733	0.0755	0.0181	1.0000	0.3157	3.1674
MAR	0.5651	0.0651	0.1097	0.0883	0.9950	0.5839	1.7041
ARUnif	0.8006	0.0506	0.0794	0.0612	1.0000	0.5225	1.9138
ARCau	0.6966	0.0566	0.0972	0.0791	1.0000	0.0678	1.6957
MCBEV	0.4300	0.1020	0.1451	0.1032	0.9100	0.4707	1.9334
ARCH	0.7984	0.0366	0.0713	0.0612	1.0000	0.5236	1.9100
AR	0.7923	0.2077	0.2155	0.0576	0.9910	0.5303	1.8687
*F b=30*							
MMFrec	0.2514	0.0514	0.0558	0.0217	1.0000	0.3638	2.7488
MAR	0.5698	0.0698	0.1244	0.1031	0.9980	0.6636	1.5040
ARUnif	0.7726	0.0226	0.0742	0.0707	1.0000	0.5914	1.6910
ARCau	0.6840	0.0440	0.1000	0.0899	0.9990	0.6467	1.5447
MCBEV	0.4379	0.1099	0.1644	0.1223	0.9500	0.5712	1.6632
ARCH	0.7804	0.0546	0.0865	0.0672	1.0000	0.5847	1.7104
AR	0.7861	0.2139	0.2230	0.0629	0.9990	0.5831	1.7132
*F b=40*							
MMFrec	0.2407	0.0407	0.0480	0.0255	1.0000	0.4125	2.4245
MAR	0.5749	0.0749	0.1345	0.1118	0.9970	0.7121	1.4000
ARUnif	0.7516	0.0016	0.0769	0.0769	1.0000	0.6417	1.5584
ARCau	0.6767	0.0367	0.1032	0.0965	0.9900	0.6870	1.4540
MCBEV	0.4495	0.1215	0.1818	0.1354	0.9720	0.6419	1.5143
ARCH	0.7646	0.0704	0.0992	0.0698	1.0000	0.6362	1.5719
AR	0.7763	0.2237	0.2335	0.0669	1.0000	0.6271	1.5947
*F b=50*							
MMFrec	0.2346	0.0346	0.0453	0.0292	1.0000	0.4617	2.1658
MAR	0.5789	0.0789	0.1403	0.1161	0.9980	0.7476	1.3349
ARUnif	0.7355	0.0145	0.0817	0.0804	1.0000	0.6849	1.4600
ARCau	0.6718	0.0318	0.1050	0.1001	0.9990	0.7207	1.3862
MCBEV	0.4625	0.1345	0.1960	0.1426	0.9810	0.7005	1.4003
ARCH	0.7508	0.0842	0.1119	0.0737	1.0000	0.6769	1.4773
AR	0.7661	0.2339	0.2440	0.0694	0.9980	0.6660	1.4985
*F b=60*							
MMFrec	0.2300	0.0300	0.0450	0.0330	0.9990	0.5061	1.9740
MAR	0.5800	0.0800	0.1450	0.1190	0.9980	0.7797	1.2800
ARUnif	0.7210	0.0290	0.0840	0.0810	1.0000	0.7212	1.3865
ARCau	0.6670	0.0270	0.1050	0.1010	0.9990	0.7522	1.3280
MCBEV	0.4720	0.1440	0.2050	0.1450	0.9930	0.7447	1.3334
ARCH	0.7390	0.0960	0.1220	0.0750	1.0000	0.7097	1.4090
AR	0.7540	0.2460	0.2550	0.0700	0.9990	0.7007	1.4257
*F b=70*							
MMFrec	0.2272	0.0272	0.0460	0.0371	0.9990	0.5530	1.8066
MAR	0.5825	0.0825	0.1447	0.1189	0.9980	0.8038	1.2417
ARUnif	0.7096	0.0404	0.0929	0.0837	1.0000	0.7506	1.3322
ARCau	0.6596	0.0196	0.1038	0.1020	0.9980	0.7781	1.2827
MCBEV	0.4814	0.1534	0.2138	0.1490	0.9930	0.7766	1.2786
ARCH	0.7265	0.1085	0.1319	0.0751	1.0000	0.7420	1.3477
AR	0.7410	0.2590	0.2686	0.0713	1.0000	0.7333	1.3637

**Table 4:** Simulation results of models MAR with $\theta =0.1$ and $\theta =0.9$, AR($\phi =0.1$) and AR($\phi =-0.1$), obtained for the runs (R), cycles (C), intervals (I), truncated (T) and K-gaps (K): mean, absolute bias (abias), root mean squared error (rmse), standard deviation (sd), the coverage proportion of the true extremal index value within the estimated confidence intervals (cov), the intervals range width (rw) and the ratio between “cov" and “rw"

	Mean	Abias	Rmse	Sd	Cov	Rw	Cov/rw
*R*							
MAR($\phi =0.9); \; \theta =0.1$	0.0941	0.0059	0.0233	0.0225	0.7020	0.1017	6.9058
MAR($\phi =0.1); \; \theta =0.9$	0.6735	0.2265	0.2415	0.0839	0.0140	0.1319	0.1062
AR($\phi =-0.1); \; \theta =1$	0.7750	0.2250	0.2426	0.0909	0.0050	0.1148	0.0435
AR($\phi =0.1); \; \theta =1$	0.7026	0.2974	0.3083	0.0812	0.0010	0.1244	0.0080
*C*							
MAR($\phi =0.9); \; \theta =0.1$	0.0913	0.0087	0.0246	0.0230	0.7700	0.1022	7.5342
MAR($\phi =0.1); \; \theta =0.9$	0.6480	0.2520	0.2722	0.1030	0.0104	0.1342	0.1043
AR($\phi =-0.1); \; \theta =1$	0.7394	0.2606	0.2868	0.1199	0.0000	0.1210	0.0000
AR($\phi =0.1); \; \theta =1$	0.6689	0.3311	0.3480	0.1070	0.0000	0.1314	0.0000
*I*							
MAR($\phi =0.9); \; \theta =0.1$	0.1010	0.0010	0.0273	0.0273	0.7920	0.1585	4.9971
MAR($\phi =0.1); \; \theta =0.9$	0.8977	0.0023	0.0675	0.0675	0.9250	0.2216	4.1737
AR($\phi =-0.1); \; \theta =1$	0.9901	0.0099	0.0222	0.0198	1.0000	0.0878	11.3901
AR($\phi =0.1); \; \theta =1$	0.9383	0.0617	0.0852	0.0587	0.9630	0.1782	5.4052
*T*							
MAR($\phi =0.9); \; \theta =0.1$	0.1340	0.0340	0.1257	0.1212	0.3500	0.1592	2.1985
MAR($\phi =0.1); \; \theta =0.9$	0.9025	0.0025	0.0469	0.0464	0.9660	0.1797	5.3756
AR($\phi =-0.1); \; \theta =1$	0.9971	0.0029	0.0113	0.0109	0.8410	0.1690	4.9763
AR($\phi =0.1); \; \theta =1$	0.9446	0.0554	0.0761	0.0531	0.8100	0.1786	4.5353
*K-gaps*							
MAR($\phi =0.9); \; \theta =0.1$	0.1034	0.0034	0.0243	0.0240	0.8790	0.0735	11.9590
MAR($\phi =0.1); \; \theta =0.9$	0.7178	0.1822	0.1929	0.0634	0.0170	0.1123	0.1513
AR($\phi =-0.1); \; \theta =1$	0.8090	0.1910	0.2047	0.0736	0.0000	0.0982	0.0000
AR($\phi =0.1); \; \theta =1$	0.7423	0.2577	0.2659	0.0658	0.0000	0.1089	0.0000

**Table 5:** Simulation results of models MAR with $\theta =0.1$ and $\theta =0.9$, AR($\phi =0.1$) and AR($\phi =-0.1$), obtained for the ( Northrop 2015) sliding blocks estimator: mean, absolute bias (abias), root mean squared error (rmse), standard deviation (sd), the coverage proportion of the true extremal index value within the estimated confidence intervals (cov), the intervals range width (rw), the ratio between “cov" and “rw" and the number of replicates where the confidence intervals couldn’t be calculated (NAs)

	Mean	Abias	Rmse	Sd	Cov	Rrw	Cov/rw	NAs
*N b=10*								
MAR($\phi =0.9); \; \theta =0.1$	0.1939	0.0939	0.0946	0.0115	0.0000	0.0249	0.0000	76
MAR($\phi =0.1); \; \theta =0.9$	0.9103	0.0103	0.0492	0.0481	0.8779	0.1591	5.5171	1
AR($\phi =-0.1); \; \theta =1$	0.9935	0.0005	0.0184	0.0172	0.9919	0.0548	18.0998	9
AR($\phi =0.1); \; \theta =1$	0.9490	0.0510	0.0660	0.0419	0.7938	0.1357	5.8484	1
*N b=20*								
MAR($\phi =0.9); \; \theta =0.1$	0.1484	0.0484	0.0505	0.0147	0.0050	0.0461	0.1084	0
MAR($\phi =0.1); \; \theta =0.9$	0.9030	0.0030	0.0655	0.0655	0.8825	0.2086	4.2302	13
AR($\phi =-0.1); \; \theta =1$	0.9823	0.0177	0.0372	0.0328	0.9621	0.1069	9.0034	51
AR($\phi =0.1); \; \theta =1$	0.9526	0.0474	0.0701	0.0516	0.8799	0.1629	5.4019	26
*N b=30*								
MAR($\phi =0.9); \; \theta =0.1$	0.1331	0.0331	0.0374	0.0173	0.4474	0.06707	7.3683	1
MAR($\phi =0.1); \; \theta =0.9$	0.8991	0.0009	0.0771	0.0771	0.8799	0.2368	3.7158	9
AR($\phi =-0.1); \; \theta =1$	0.9721	0.0279	0.0520	2 0.0441	0.9615	0.1463	6.5733	38
AR($\phi =0.1); \; \theta =1$	0.9504	0.0496	0.0777	0.0599	0.9229	0.1902	4.8521	14
*N b=40*								
MAR($\phi =0.9); \; \theta =0.1$	0.1257	0.0257	0.0324	0.0196	0.7296	0.0707	10.3239	5
MAR($\phi =0.1); \; \theta =0.9$	0.8952	0.0048	0.0863	0.0862	0.8463	0.2653	3.1899	76
AR($\phi =-0.1); \; \theta =1$	0.9641	0.0359	0.0646	0.0538	0.9287	0.1832	5.0686	159
AR($\phi =0.1); \; \theta =1$	0.9475	0.0525	0.0853	0.0673	0.8890	0.2108	4.2171	126
*N b=50*								
MAR($\phi =0.9); \; \theta =0.1$	0.1715	0.0215	0.0309	0.0222	0.8141	0.0798	10.2055	10
MAR($\phi =0.1); \; \theta =0.9$	0.8918	0.0082	0.0951	0.0948	0.8313	0.2851	2.9155	117
AR($\phi =-0.1); \; \theta =1$	0.9574	0.0426	0.0745	0.0612	0.9272	0.2149	4.3135	190
AR($\phi =0.1); \; \theta =1$	0.9426	0.0574	0.0938	0.0743	0.8862	0.2444	3.6256	165
*N b=60*								
MAR($\phi =0.9); \; \theta =0.1$	0.1185	0.0185	0.0308	0.0246	0.8900	0.0905	9.8349	0
MAR($\phi =0.1); \; \theta =0.9$	0.8860	0.0140	0.1038	0.1030	0.8447	0.3107	2.7188	28
AR($\phi =-0.1); \; \theta =1$	0.9503	0.0497	0.0848	0.0688	0.9263	0.2250	4.1170	64
AR($\phi =0.1); \; \theta =1$	0.9376	0.0624	0.1025	0.0814	0.9057	0.2533	3.5762	56
*N b=70*								
MAR($\phi =0.9); \; \theta =0.1$	0.1170	0.0170	0.0321	0.0273	0.8516	0.0944	9.0179	16
MAR($\phi =0.1); \; \theta =0.9$	0.8838	0.0162	0.1101	0.1090	0.8328	0.3295	2.5274	109
AR($\phi =-0.1); \; \theta =1$	0.9453	0.0547	0.0929	0.0751	0.9165	0.2651	3.4565	198
AR($\phi =0.1); \; \theta =1$	0.9344	0.0656	0.1093	0.0875	0.8847	0.2835	3.1203	159

**Table 6:** Simulation results of models MAR with $\theta =0.1$ and $\theta =0.9$, AR($\phi =0.1$) and AR($\phi =-0.1$), obtained for the Ferreira and Ferreira (2022) sliding blocks estimator: mean, absolute bias (abias), root mean squared error (rmse), standard deviation (sd), the coverage proportion of the true extremal index value within the estimated confidence intervals (cov), the intervals range width (rw), the ratio between “cov" and “rw" and the number of replicates where the confidence intervals couldn’t be calculated (NAs)

	Mean	Abias	Rmse	Sd	Cov	Rw	Cov/rw
*F b=10*							
MAR($\phi =0.9); \; \theta =0.1$	0.2160	0.1160	0.1206	0.0333	0.0750	0.1854	0.4046
MAR($\phi =0.1); \; \theta =0.9$	0.8695	0.0305	0.0466	0.0352	1.0000	0.3887	2.5730
AR($\phi =-0.1); \; \theta =1$	0.9216	0.0784	0.0811	0.0207	1.0000	0.3285	3.0438
AR($\phi =0.1); \; \theta =1$	0.9069	0.0931	0.0958	0.0230	1.0000	0.3472	2.8804
*F b=20*							
MAR($\phi =0.9); \; \theta =0.1$	0.1935	0.0935	0.1056	0.0491	0.6500	0.2345	2.7717
MAR($\phi =0.1); \; \theta =0.9$	0.8409	0.0591	0.0746	0.0455	1.0000	0.4858	2.0585
AR($\phi =-0.1); \; \theta =1$	0.8838	0.1162	0.1198	0.0294	1.0000	0.4419	2.2627
AR($\phi =0.1); \; \theta =1$	0.8776	0.1224	0.1261	0.0306	1.0000	0.4497	2.2237
*F b=30*							
MAR($\phi =0.9); \; \theta =0.1$	0.2024	0.1024	0.1208	0.0640	0.7600	0.2957	2.5702
MAR($\phi =0.1); \; \theta =0.9$	0.8185	0.0915	0.0963	0.0513	1.0000	0.5541	1.8047
AR($\phi =-0.1); \; \theta =1$	0.8563	0.1437	0.1483	0.0367	1.0000	0.5191	1.9266
AR($\phi =0.1); \; \theta =1$	0.8527	0.1473	0.1517	0.0363	1.0000	0.5229	1.9125
*F b=40*							
MAR($\phi =0.9); \; \theta =0.1$	0.2179	0.1179	0.1403	0.0761	0.8120	0.3637	2.2324
MAR($\phi =0.1); \; \theta =0.9$	0.7990	0.1010	0.1156	0.0563	1.0000	0.6087	1.6454
AR($\phi =-0.1); \; \theta =1$	0.8332	0.1668	0.1720	0.0421	1.0000	0.5780	1.7300
AR($\phi =0.1); \; \theta =1$	0.8328	0.1677	0.1719	0.0399	1.0000	0.5788	1.7276
*F b=50*							
MAR($\phi =0.9); \; \theta =0.1$	0.2360	0.1363	0.1611	0.0859	0.8400	0.4395	1.9113
MAR($\phi =0.1); \; \theta =0.9$	0.7843	0.1157	0.1299	0.0591	1.0000	0.6499	1.5387
AR($\phi =-0.1); \; \theta =1$	0.8144	0.1856	0.1316	0.0473	1.0000	0.6250	1.5999
AR($\phi =0.1); \; \theta =1$	0.8127	0.1873	0.1929	0.0459	1.0000	0.6285	1.5912
*F b=60*							
MAR($\phi =0.9); \; \theta =0.1$	0.2566	0.1566	0.1837	0.0960	0.8670	0.5148	1.6840
MAR($\phi =0.1); \; \theta =0.9$	0.7674	0.1326	0.1475	0.0646	1.0000	0.6913	1.4466
AR($\phi =-0.1); \; \theta =1$	0.7953	0.2047	0.2104	0.0488	1.0000	0.6707	1.4910
AR($\phi =0.1); \; \theta =1$	0.7961	0.2039	0.2100	0.0504	1.0000	0.6686	1.4957
*F b=70*							
MAR($\phi =0.9); \; \theta =0.1$	0.2769	0.1769	0.2060	0.1055	0.8960	0.5931	1.5106
MAR($\phi =0.1); \; \theta =0.9$	0.7538	0.1472	0.1616	0.0667	1.0000	0.7231	1.3830
AR($\phi =-0.1); \; \theta =1$	0.7788	0.2212	0.2278	0.0546	1.0000	0.7053	1.4178
AR($\phi =0.1); \; \theta =1$	0.7768	0.2232	0.2298	0.0545	1.0000	0.7092	1.4101

**Table 7:** Coverage proportion of the true extremal index value within the estimated bootstrap confidence intervals (cov), the intervals range width (rw) and the ratio between “cov" and “rw", considering automated choice of block length in Politis and White (2004) and Patton et al. (2009), within runs (R), cycles (C) and truncated (T) estimators

	R			C			T		
	Cov	Rw	Cov/rw	Cov	Rw	Cov/rw	Cov	Rw	cov/rw
MMFrec	0.9490	0.0826	11.4905	0.7820	0.0843	9.2772	0.2850	0.1203	2.3685
MAR	0.8190	0.1480	5.5354	0.8120	0.1478	5.4939	0.7670	0.1635	4.6920
MAR($\phi =0.9); \; \theta =0.1$	0.8700	0.1050	8.2818	0.9080	0.1341	0.2088	0.6730	0.1814	5.3630
MAR($\phi =0.1); \; \theta =0.9$	0.0280	0.1272	0.2202	0.0280	0.1048	8.6648	0.9730	0.1480	4.5471
ARUnif	0.7190	0.1083	6.6377	0.4890	0.1134	4.3107	0.0185	0.0778	0.2381
ARCau	0.3310	0.1618	2.0455	0.1830	0.1628	1.1243	0.3273	0.2009	1.6291
MCBEV	0.7600	0.1866	4.0721	0.7300	0.1836	3.9768	0.4640	0.2048	2.2652
ARCH	0.6530	0.1187	5.5014	0.6250	0.1191	5.2458	0.0790	0.1471	0.5371
AR	0.0040	0.1532	0.0261	0.0040	0.1580	0.0253	0.0040	0.1814	0.0221
AR($\phi =0.1); \; \theta =1$	0.0010	0.1244	0.0080	0.0010	0.1319	0.0076	0.9460	0.1813	5.2166
AR($\phi =-0.1); \; \theta =1$	0.0040	0.1178	0.0339	0.0040	0.1243	0.0322	0.9570	0.1732	5.5241

## Application

**Fig. 5:**
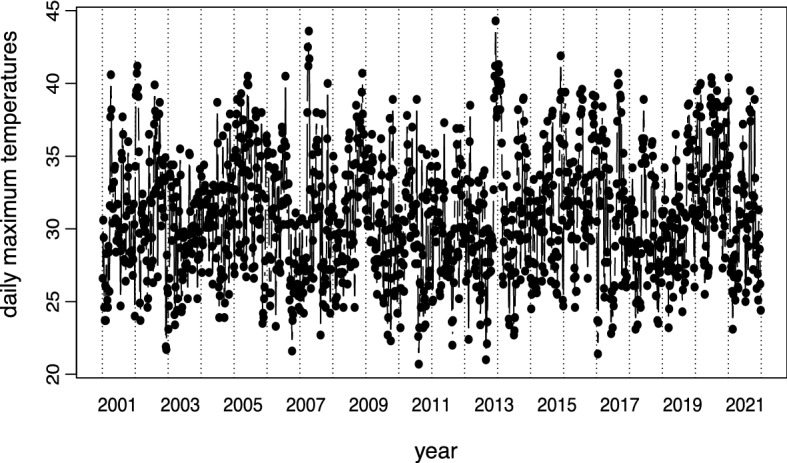
Daily maximum temperature (in Celsius degrees) in the months of July and August, from 2001 to 2021, at the climatologic station Abrantes in the center of Portugal

**Fig. 6:**
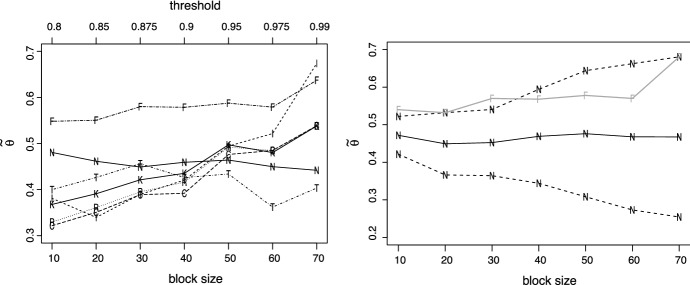
Left: Estimates of $\theta$ for daily maximum temperatures obtained with the runs (R), cycles (C), intervals (I), truncated (T) and *K*-gaps (K) estimators using thresholds corresponding to quantiles 0.8, 0.875, 0.9, 0.95, 0.975, 0.99, under the validity of $D^{(3)}$ condition derived from IMT method, and estimates of $\theta$ obtained from sliding blocks ${\tilde{\theta }}^{(F)}_{sl}$ and ${\tilde{\theta }}^{(N)}_{sl}$, for block sizes $b=10,20,...,70$; Right: Sliding blocks estimates from ${\tilde{\theta }}^{(F)}_{sl}$ and ${\tilde{\theta }}^{(N)}_{sl}$, for block sizes $b=10,20,...,70$, and ${\tilde{\theta }}^{(N)}_{sl}$ lower and upper 95% confidence bands

We consider the daily maximum temperatures in the months of July and August, from 2001 to 2021, collected at the climatologic station Abrantes in center of Portugal (in Celsius degrees) available at “SNIRH: Sistema Nacional de Informação dos Recursos Hídricos".^1^ The data is plotted in Fig. 5, in which successive high temperatures are seen, which are typical in this inland city. The results in Table 8 are obtained through the application of IMT procedure, leading to threshold 37.7 and $K=2$. Thus we assume $D^{(3)}(u_n)$ dependence condition. Figure 6 represents the estimates of ${\tilde{\theta }}^{(R)}$, ${\tilde{\theta }}^{(I)}$, ${\tilde{\theta }}^{(C)}$. ${\tilde{\theta }}^{(T)}$ and ${\tilde{\theta }}^{(K)}$, for thresholds corresponding to quantiles from 0.8 to 0.99. The estimators give quite close results to each other, except the sliding blocks ${\tilde{\theta }}^{(F)}$ with larger values (Fig. 6, left). However, most of ${\tilde{\theta }}^{(F)}$ estimates are within the Northrop sliding blocks confidence bands (Fig. 6, right), which we use as reference according to the simulation study findings in Sect. 3. Our guess is that possible values for $\theta$ range between 0.4 and 0.55.

**Table 8:** Estimation results obtained for daily maximum temperatures when applying the runs (R), cycles (C), intervals (I), truncated (T) and *K*-gaps (K) estimators based on IMT method which leads to the validity of $D^{(3)}$ condition and the choice of threshold 37.7

	Estimate	Lower	Upper	Rw
R	0.4190	0.3373	0.5670	0.2297
C	0.4095	0.3550	0.5589	0.2040
I	0.4423	0.3293	0.6456	0.3162
T	0.4474	0.3796	0.6328	0.2532
K	0.4340	0.3595	0.5128	0.1533

## Discussion

The extremal index is a very important measure in inferring extreme values of time series. In addition to affecting the limiting law behavior of the maximum, it is also associated with a clustering effect of exceedances of high values that can have harmful consequences. The estimation of the extremal index is a long-standing subject in extreme value theory, and it still attracts the attention of researchers. Here we present a study that covers several estimators, from the classic runs estimator to the most recent methodologies in block maxima, such as ( Ferreira and Ferreira (2022)) work resorting the theory of bivariate extremes. This new approach is still at an early stage, and there is room to improve both the estimation methodology that requires the generation of auxiliary samples and the analysis of the asymptotic behavior of the estimator and obtaining confidence intervals. Finding the asymptotic variance of the extremal index estimators is a great challenge given the context of serial dependence. Thus the resampling methodologies appear as good alternatives. However the choice of bootstrap block length is a critical point of estimation. A careful analysis is still needed to ameliorate in practice the coverage probabilities which proved to be poor in some cases. The great advanced in computing capabilities that we are currently witnessing opens up good prospects for the implementation and development of techniques such as bootstrap or jackknife. The area of machine learning is also a whole new horizon waiting to be explored.

## Footnotes

1 https://snirh.apambiente.pt/index.php?idMain=1 &idItem=1.6.
